# Determinants and constraints to household-level animal source food consumption in rural communities of Ethiopia

**DOI:** 10.1017/jns.2021.52

**Published:** 2021-08-09

**Authors:** Alemneh Kabeta Daba, Mary Murimi, Kebede Abegaz, Dejene Hailu

**Affiliations:** 1 School of Nutrition, Food Science and Technology, College of Agriculture, Hawassa University, Hawassa, Ethiopia; 2 School of Nursing, College of Medicine and Health Sciences, Hawassa University, Hawassa, Ethiopia; 3 Department of Nutritional Sciences, College of Human Sciences, Texas Tech University, Lubbock, TX, USA; 4 School of Public Health, College of Medicine and Health Sciences, Hawassa University, Hawassa, Ethiopia

**Keywords:** Animal source foods, Consumption frequency, Constraints and determinants, Households in Ethiopia, AOR, adjusted odds ratio, ASF, animal source foods, BMI, body mass index, ETB, Ethiopian Birr; ha, hectare, IQR, Inter-quartile Range, SD, Standard Deviation, USA, United States of America, USAID, United States Agency for International Development, USD, United States' Dollar

## Abstract

Animal source foods (ASF) contain quality nutrients important for growth, development, immunity and behavioural outcomes. Plant-based foods also provide the nutrients, but with lower bioavailability than ASF. Evidence on household-level ASF consumption frequency, constraints and determinants are limited for Ethiopia. Therefore, the present study aimed to assess the consumption frequency of ASF and to identify determinants and constraints among rural households in Ethiopia. A cross-sectional study was conducted in 422 households. The consumption frequency of ASF was assessed using a food frequency screener over 30 days. Twelve statements with Likert scale responses were used to identify constraints to ASF consumption. Ordinal logistic regression was used to identify determinants of ASF consumption. About a quarter (26 %) of the households consumed milk one to two times per week. One out of five households consumed eggs one to two times per week (20 %) or one to two times per month (19 %). Poultry and meat were never consumed by 92 and 60 % of the households, respectively. Unavailability, unaffordability, consumption tradition and income generation priority were constraints identified. Food insecurity, livestock ownership, income, family size and women's education were associated (*P* < 0⋅05) with selected ASF consumption. Rural households in Ethiopia did not consume ASF on regular basis. Poor socio-demographic and economic conditions as determined by food insecurity, property ownership, income, educational achievement, family size and ASF unavailability and unaffordability contributed to the lower consumption frequency of ASF by households in rural Ethiopia. Nutrition policies and programmes should focus on nutrition-sensitive agricultural extension, livelihood improvement and women empowerment interventions integrated with nutrition education to improve ASF consumption in rural settings.

## Introduction

Malnutrition is a significant public health concern. Globally, out of 676 million children underfive^(1)^, 155 million are stunted and 52 million are wasted^(2,3)^. Worldwide, almost three million children die before the age of 5 years and 45 % of those deaths are attributed to malnutrition^(4)^. Africa is home to about fifty-nine million stunted and fourteen million wasted children in the world. Regionally, countries located in East Africa share 4⋅1 % of wasting and 24 % of stunting^(3)^.

Undernutrition can be manifested as either growth failure or micronutrient deficiency, and a multitude of factors contribute to undernutrition^(5)^. For example, low animal source food (ASF) consumption has been reported to increase the risks of being undernourished^(6–8)^. ASF are excellent sources of quality macro- and micro-nutrients^(9)^ including all of the essential amino acids^(10)^, zinc, iron, calcium, selenium, vitamin A, vitamin B_12_^(11)^ and vitamin D^(12)^. Having access to such foods is vital for bone health, growth, healthy blood cell production, immunity, neurological function and behavioural outcomes^(13–15)^. Moreover, the addition of small quantities of ASF in a diet can improve the nutritional performance of plant-based diets and consumers’ nutritional status^(15–17)^. However, there are still many communities throughout the world that have poor or minimal access to ASF^(18)^.

Ethiopia has a high burden of malnutrition. According to the 2016 Ethiopian Demographic and Health Survey, within half a decade small decrements in stunting (44–38 %), wasting (12–10 %) and underweight (29–24 %) for children underfive were reported. In addition, more than half (56 %) of children underfive suffer from iron deficiency anaemia^(19)^. Ethiopia is committed to ending hunger and dramatically reducing stunting by 2030^(20)^ and emphasised the need for a moderate increase in ASF consumption^(21)^. Ethiopians consume on average one-tenths as much meat as people in developed countries. Dietary diversity and other food consumption pattern assessments also reported that a limited proportion of diets in Ethiopia contain ASF. For example, according to a survey on household-level dietary diversity, diets in more than 90 % of households were cereal-dominated with little inclusion of ASF^(22)^. Evidence from the Ethiopian National Food Consumption Survey^(23)^ and small area assessments reported that only a small proportion (11–16 %)^(24–27)^ of children met minimum dietary diversity recommendations^(28)^, and investigations documented ASF as the most limiting food group^(29,30)^.

Women and children are more vulnerable to malnutrition as compared to the general population and it is estimated that almost 30 % of Ethiopian women of reproductive age had a body mass index (BMI) less than 18⋅5 kg/m^2(31)^. A study^(32)^ in Ethiopia with the majority (71 %) of female participants also estimated that the intakes of nutrients available from ASF were below the recommended levels for protein (11 %), calcium (90 %) and vitamin A (100 %). Furthermore, almost a quarter (23 %) of women in the country had iron deficiency anaemia^(19)^ for which ASF have been found to be beneficial^(33)^. According to a study^(34)^ from northern Ethiopia, the proportion of lactating women who ate ASF was also low, with 17 % consuming meat and fish, 18 % eggs and 12 % dairy products.

While ASF may protect against undernutrition, research on dietary patterns in Ethiopia has not addressed household-level ASF consumption patterns^(22,25–27,29,30)^. Furthermore, those published studies have not investigated constraints to and determinants of ASF consumption in the country. Hence, the purpose of the present study was to determine household- or family-level dietary behaviours related to the consumption of ASF and to identify facilitators, constraints to and determinants of ASF consumption. The findings of the present study can be a base to strategize and realise far-reaching food-based nutrition interventions and to reduce the magnitude of undernutrition among families in Ethiopia^(35,36)^.

## Materials and methods

### Study area and design

This cross-sectional study was conducted in rural kebeles (the lowest unit in Ethiopia's administrative structure) from the Milkshed region of Hawassa, districts of Oromia and Sidama regional states. The milkshed includes Arsi Negelle (Oromia), Dale, Wondo Genet and other districts (Sidama)^(37)^.

### Sample size and sampling

Sample size was calculated using a single population proportion formula^(38)^ considering 50 % proportion (no findings on household-level ASF consumption frequency to base the estimate on), a 95 % confidence level, 0⋅05 of *α* and 10 % non-response rate. Data were collected from 422 households selected using a simple random sampling technique. Four kebeles were randomly selected from each district (twelve kebeles in total). An equal proportion allocation was applied to draw sample size from each of the selected kebeles. All the respondents were women who were responsible for food preparation for the family.

### Data collection

Data were collected by individual interviews using a pre-tested questionnaire. The pre-test was done in districts from Oromia and Sidama regional states, but other than districts where the actual data were collected. The tool was constituted of four parts: (1) socio-demographic and economic characteristics, (2) household food-insecurity access scale^(39)^, (3) selected ASF consumption frequency screener and (4) facilitators and constraints to the consumption of specific ASF questionnaire in Likert scales.

The ASF consumption frequency screener for household- (family-) level selected ASF consumption during the 30 days had closed-ended response options. These were ‘greater than once per day’, ‘once per day’, ‘three to six times per week’, ‘one to two times per week’, ‘one to two times per fortnight’, ‘one to two times per month’ and ‘never consumed in the last month’ for consumption frequencies of meat, poultry, fish, eggs, milk and milk products.

Data on facilitators and constraints to the consumption of ASF were collected using predefined statements with five-level Likert scale response options. The statements focused on quality, availability, affordability, price of ASF as compared to plant-based food, effort involved to prepare food products from the specific ASF, ASF storage facilities, health beliefs about specific ASF, medical concern related to the consumption of ASF, ASF consumption tradition during childhood, religious restrictions on the consumption of ASF, fasting restrictions to ASF consumption and income generation priority from livestock rearing. The twelve predefined statements included items such as ‘The quality of meat/fish/eggs/milk/milk products is important for me’, ‘I am able to afford meat/fish/eggs/milk/milk products’, ‘Eating plant-based foods is more affordable for us than eating meat/fish/eggs/milk/milk products’ and others. All statements were provided with response options of-‘strongly agree/ slightly agree/ neither agree nor disagree/ slightly disagree/ strongly disagree.’

The data collectors attended 3 days training on the basics of data collection methods, research ethics and the purpose of the present study. The principal investigator supervised data collection and reviewed the questionnaires for completeness.

### Data analysis

Data were coded and entered into SPSS version 20 for Windows and cleaned. Frequencies, proportions, mean or median scores and standard deviations (sd) or inter-quartile ranges (IQR) were computed. After checking for the existence of multicollinearity, predictors of specific ASF consumption frequency were identified with the application of cumulative odds ordinal logistic regression with proportional odds model at significance set at *P* < 0⋅05. The regression analysis was not done for fish because it was consumed by a small proportion of households in 30 days before the survey.

### Ethical consideration

The present study was conducted according to the guidelines laid down in the Declaration of Helsinki, and all procedures involving human subjects were approved by the Institutional Review Board of Hawassa University (Ref. No.: IRB/027/10). Verbal informed consent was obtained from all subjects before data collection. Verbal consent was witnessed and formally recorded.

## Results

### Socio-demographic and economic characteristics

Almost all of the women were married (96 %) and 81 % were homemakers. Regarding education, some of the respondents (34 %) had completed from grades one to five, while 43 % had completed from grades six to twelve. Respondents’ mean (sd) age was 25⋅55 (4⋅96) years. The majority of the participants identified themselves as Sidama (61 %) by ethnicity and protestant (67 %) (Table 1).

**Table 1.** Socio-demographic and economic characteristics of households and respondents from Hawassa Milkshed, Ethiopia (*N* 422)

Variables	*N*	%
Respondents’ characteristics		
Educational status		
Never attended school	87	20⋅6
Grades 1–5	144	34⋅3
Grades 6–12	179	42⋅6
College and above	12	2⋅8
Marital status		
Married	407	96⋅4
Single	15	3⋅5
Age (in completed years)		
≤18	25	5⋅9
19–30	335	79⋅4
≥31	62	14⋅6
Ethnicity		
Sidama	257	60⋅9
Oromo	132	31⋅3
Wolayta	13	3⋅1
Others	20	4⋅7
Religion		
Protestant	283	67⋅0
Muslim	117	27⋅7
Orthodox	14	3⋅3
Catholic	8	1⋅9
Occupation		
Homemaker	343	81⋅3
Farmer	31	7⋅3
Merchant	30	7⋅1
Others	14	3⋅7
Households’ characteristics		
Estimated annual income in ETB/US dollar ($)		
≤5000/$163	182	46⋅4
5001–15 000/$164–492	131	33⋅8
15 001–30 000/$492–983	57	14⋅7
≥30 001/$984	34	8⋅8
Head		
Woman	17	4⋅0
Man	396	93⋅9
Grandparent/s	9	2⋅1
Who decides on daily household needs?		
Woman	246	58⋅3
Man	39	9⋅2
Both husband and wife	130	30⋅8
Others	7	1⋅7
Who decides how household income used?		
Woman	74	17⋅5
Man	90	21⋅3
Both husband and wife	251	59⋅5
Others	7	1⋅7
Family size		
≤3	73	17⋅3
4–6	240	56⋅9
≥7	109	25⋅8
Domestic animal ownership		
Ox	99	23⋅4
Cow	181	42⋅8
Goat	50	11⋅7
Sheep	39	9⋅2
Chicken	175	41⋅4
Agriculture land ownership (ha)		
No agriculture land	21	5
<1	197	46⋅7
1–2	161	38⋅2
>2	43	10⋅2
Food insecurity		
Secure	196	46⋅4
Insecure	226	53⋅6

More than half (57 %) of the households had four to six family members, and almost all (94 %) were male-headed. The median (IQR) annual household income was 6650 (3000, 15 000) Ethiopian Birr (ETB) [1USD ≈ Birr 30⋅50]. This monetary income is minor in the rural family settings, where their major living expenditures are derived in-kind from their own agricultural produce, and their lifestyle is associated with low-cost family labour and with natural utilities like fuel/energy, water and housing materials. The majority (80 %) of the households earned less than 15 000 ETB or $491 annually on top of farm goods and services that make their major living. Decisions on household needs were made by the women for 58 % of the households or by both husband and wife for 31 % of the households. Almost all (95 %) households owned different amounts of agricultural land with 48 % of the households owning less than 1 ha. Concerning domestic animal ownership, about 43 % of households owned cows and 41 % owned chickens. More than half (54 %) of the households were food insecure (Table 1).

### Consumption frequency of animal source foods

Poultry products were not consumed in 92 % of households during the month before the survey. Meat from sheep or lamb, goat, beef or cattle and other large animals was consumed one to two times per month by 26 % of the households. Fish products were only consumed by 3 % of the households.

Milk was consumed once per day by less than one-quarter of the households (21 %) and by a quarter (26 %) one to two times per week. Milk products like yogurt and cheese were consumed one to two times per month by 15 % of the households. Eggs were consumed one to two times per week by 20 % and one to two times per month by 19 % of households (Table 2).

**Table 2.** Consumption frequency of ASF by households from Hawassa Milkshed, Ethiopia (*N* 422)

Animal source food	Consumption frequency	*N*	%
Poultry	More than once per day to one to two times per fortnight	11	2⋅6
	One to two times per month	24	5⋅7
	Never consumed	387	91⋅7
Meat (sheep/lamb, goat, beef/cattle and any other animals)	More than once per day to one to two times per week	42	10⋅0
	One to two times per fortnight	17	4⋅0
	One to two times per month	110	26⋅1
	Never consumed	253	60⋅0
Fish products	More than once per day to one to two times per month	11	2⋅6
	Never consumed	411	97⋅4
Eggs	More than once or once per day	12	2⋅8
	Three to six times per week	32	7⋅6
	One to two times per week	86	20⋅4
	One to two times per fortnight	20	4⋅7
	One to two times per month	81	19⋅2
	Never consumed	191	45⋅3
Milk	More than once or once per day	90	21⋅3
	Three to six times per week	47	11⋅1
	One to two times per week	108	25⋅6
	One to two times per fortnight	22	5⋅2
	One to two times per month	76	18⋅0
	Never consumed	79	18⋅7
Milk products (yogurt, cheese, whey, etc.)	More than once or once per day	18	4⋅3
	Three to six times per week	20	4⋅7
	One to two times per week	30	7⋅1
	One to two times per fortnight	24	5⋅7
	One to two times per month	66	15⋅6
	Never consumed	264	62⋅6

### Facilitators to the consumption of animal source foods

Nearly all of the study participants (97 %) agreed with the statement ‘The quality of meat is important for me’, and 98 % agreed that eating meat is good for their health. About 64 % of the participants did not agree with the statement ‘It takes a lot of effort to prepare and cook meat’; and 70 % did not perceive lack of proper meat storage technologies as a barrier to meat consumption. The majority of the participants reported that they did not have religious prohibition on the consumption of meat (91 %), eggs (91 %) and dairy products (93 %). Nearly all of the respondents (92 % for meat, 94 % for egg and 94 % for dairy) disagreed with the statement ‘I don't eat meat/egg/dairy products when I am fasting.’

About two-thirds of the participants reported that they can afford eggs (60 %), and the majority reported that eggs are available (78 %) where they shop. Lack of proper egg storage facility did not prevent the majority of the study participants (76 %) from egg consumption.

The majority of participants (76 %) reported that milk and milk products were regular components of their diet while growing up as children. About 82 % of respondents agreed that milk and milk products are available where they usually shop, and more than two-thirds (70 %) agreed that they could afford milk and milk products (Supplementary Table S1).

### Constraints to the consumption of animal source foods

Most of the participants (70 %) agreed that it is difficult to get meat where they usually shop, while the majority of them (80 %) indicated that they cannot afford meat (Supplementary Table S1). The majority reported that plant-based food products were more affordable than meat (92 %), eggs (93 %), dairy (90 %) and fish (91 %). About half (50 % for meat and 57 % for fish) of the participants disagreed with the statement ‘Meat or fish was a regular component of my diet while growing up.’ Almost all of the respondents (99 %) indicated that fish was not available where they usually shop, while more than half of the participants (58 %) reported that they could not afford fish (Supplementary Tables S1 and S2).

### Determinants of household-level animal source food consumption frequency

Household food insecurity was significantly associated with consumption frequencies of all ASF types. Food-insecure households were less likely to consume poultry (adjusted odds ratio (AOR) 0⋅35, *P* 0⋅035), meat (AOR 0⋅25 *P* < 0⋅001), eggs (AOR 0⋅44, *P* < 0⋅001), milk (AOR 0⋅66, *P* 0⋅035) and milk products (AOR 0⋅52, *P* 0⋅005) than food-secure households (Tables 3 and 4).

**Table 3.** Ordinal logistic regression analysis on predictors of poultry, meat and egg consumption frequencies by households in Hawassa Milkshed, Ethiopia (*N* 422)

Characteristics	Poultry		Meat		Eggs	
	AOR (95 % CI)	*P*	AOR (95 % CI)	*P*	AOR (95 % CI)	*P*
Food Insecurity						
Insecure	0⋅35 (0⋅13, 0⋅93)	0⋅035	0⋅25 (0⋅16, 0⋅40)	0⋅000	0⋅44 (0⋅29, 0⋅66)	0⋅000
Secure	1		1		1	
Cash crops production						
No	0⋅40 (0⋅11, 1⋅46)	0⋅168	0⋅98 (0⋅46, 2⋅09)	0⋅954	0⋅92 (0⋅46, 1⋅86)	0⋅822
Yes	1		1		1	
Chicken ownership						
No	0⋅70 (0⋅28, 1⋅71)	0⋅43	0⋅90 (0⋅57, 1⋅43)	0⋅658	0⋅33 (0⋅22, 0⋅50)	0⋅000
Yes	1		1		1	
Donkey ownership						
No	0⋅25 (0⋅08, 0⋅78)	0⋅017	0⋅38 (0⋅18, 0⋅77)	0⋅008	0⋅55 (0⋅29, 1⋅03)	0⋅06
Yes	1		1		1	
Sheep ownership						
No	2⋅80 (0⋅62, 13⋅53)	0⋅176	0⋅97 (0⋅48, 1⋅96)	0⋅929	0⋅97 (0⋅51, 1⋅88)	0⋅938
Yes	1		1		1	
Goat ownership						
No	1⋅24 (0⋅37, 4⋅18)	0⋅726	1⋅58 (0⋅77, 3⋅27)	0⋅216	0⋅86 (0⋅47, 1⋅59)	0⋅636
Yes	1		1		1	
Cow ownership						
No	1⋅36 (0⋅51, 3⋅63)	0⋅544	1⋅24 (0⋅76, 2⋅02)	0⋅389	0⋅99 (0⋅63, 1⋅53)	0⋅947
Yes	1		1		1	
Ox ownership						
No	0⋅61 (0⋅23, 1⋅64)	0⋅324	1⋅56 (0⋅88, 2⋅77)	0⋅132	0⋅86 (0⋅52, 1⋅41)	0⋅541
Yes	1		1		1	
Women's educational status						
No education	0⋅1 (0⋅01, 1⋅06)	0⋅056	0⋅51 (0⋅14, 1⋅88)	0⋅311	0⋅20 (0⋅06, 0⋅71)	0⋅012
Grades 1–5	0⋅22 (0⋅03, 1⋅70)	0⋅148	0⋅94 (0⋅27, 3⋅22)	0⋅918	0⋅50 (0⋅15, 1⋅63)	0⋅248
Grades 6–10	0⋅15 (0⋅02, 1⋅12)	0⋅064	0⋅79 (0⋅23, 2⋅70)	0⋅707	0⋅68 (0⋅21, 2⋅19)	0⋅517
Higher education	1		1		1	
Agriculture land ownership and size (ha)						
No land	4⋅29 (0⋅23, 80⋅12)	0⋅33	1⋅67 (0⋅44, 6⋅28)	0⋅45	1⋅66 (0⋅48, 5⋅74)	0⋅427
<1	1⋅75 (0⋅33, 9⋅33)	0⋅511	0⋅49 (0⋅22, 1⋅06)	0⋅071	0⋅59 (0⋅28, 1⋅23)	0⋅158
1–2	1⋅63 (0⋅34, 7⋅80)	0⋅542	0⋅65 (0⋅32, 1⋅33)	0⋅239	0⋅75 (0⋅38, 1⋅5)	0⋅421
>2	1		1		1	
Family size						
≥7	0⋅11 (0⋅03, 0⋅45)	0⋅002	0⋅38 (0⋅19, 0⋅77)	0⋅007	0⋅60 (0⋅32, 1⋅14)	0⋅117
4–6	0⋅34 (0⋅13, 0⋅89)	0⋅029	0⋅59 (0⋅34, 1⋅03)	0⋅064	0⋅57 (0⋅34,0⋅95)	0⋅031
≤3	1		1		1	
Households estimated annual income in ETB						
≤5000	0⋅27 (0⋅07, 1⋅08)	0⋅064	0⋅19 (0⋅08, 0⋅42)	0⋅000	0⋅38 (0⋅18, 0⋅82)	0⋅013
5001–15 000	0⋅66 (0⋅18, 2⋅38)	0⋅524	0⋅50 (0⋅23, 1⋅11)	0⋅088	0⋅92 (0⋅43, 1⋅97)	0⋅829
15 001–30 000	0⋅86 (0⋅20, 3⋅77)	0⋅845	0⋅83 (0⋅35, 1⋅96)	0⋅672	0⋅99 (0⋅43, 2⋅29)	0⋅997
≥30 001	1		1		1	
Respondents’ religion						
Orthodox/catholic	0⋅77 (0⋅07, 9⋅03)	0⋅835	1⋅28 (0⋅50, 3⋅25)	0⋅606	2⋅37 (0⋅99, 5⋅68)	0⋅053
Muslim	3⋅19 (0⋅86, 11⋅79)	0⋅835	0⋅53 (0⋅24, 1⋅16)	0⋅113	2⋅87 (1⋅44, 5⋅71)	0⋅003
Protestant	1		1		1	

AOR, adjusted odds ratio; ha, hectare; ETB, Ethiopian Birr.

Maximum Variance inflation factor: poultry = 2⋅08, meat = 2⋅08 and eggs = 2⋅08; pseudo *R*^2^: poultry = 0⋅32, meat = 0⋅28 and eggs = 0⋅34.

**Table 4.** Ordinal logistic regression analysis on predictors of milk and milk products consumption frequencies by households in Hawassa Milkshed, Ethiopia (*N* 422)

Characteristics	Milk		Milk products (yogurt, cheese, whey, etc.)	
	AOR (95 % CI)	*P*	AOR (95 % CI)	*P*
Household food insecurity				
Insecure	0⋅66 (0⋅45, 0⋅98)	0⋅035	0⋅52 (0⋅33, 0⋅83)	0⋅005
Secure	1		1	
Cash crops production: coffee, khat, etc.				
No	0⋅93 (0⋅49, 1⋅74)	0⋅813	3⋅01 (1⋅46, 6⋅20)	0⋅003
Yes	1		1	
Chicken ownership				
No	0⋅75 (0⋅51, 1⋅11)	0⋅149	1⋅10 (0⋅69, 1⋅76)	0⋅684
Yes	1		1	
Donkey ownership				
No	0⋅91 (0⋅49, 1⋅70)	0⋅768	1⋅01 (0⋅53, 1⋅93)	0⋅979
Yes	1		1	
Sheep ownership				
No	0⋅63 (0⋅33, 1⋅21)	0⋅169	0⋅44 (0⋅22, 0⋅90)	0⋅024
Yes	1		1	
Goat ownership				
No	1⋅5 (0⋅83, 2⋅72)	0⋅182	0⋅90 (0⋅47, 1⋅71)	0⋅742
Yes	1		1	
Cow ownership				
No	0⋅42 (0⋅28, 0⋅64)	0⋅000	0⋅35 (0⋅21, 0⋅58)	0⋅000
Yes	1		1	
Ox ownership				
No	0⋅8 (0⋅49, 1⋅31)	0⋅372	0⋅95 (0⋅55, 1⋅64)	0⋅856
Yes	1		1	
Women's educational status				
No education	0⋅29 (0⋅08, 1⋅01)	0⋅052	0⋅61 (0⋅16, 2⋅36)	0⋅471
Grades 1–5	0⋅37 (0⋅11, 1⋅24)	0⋅107	1⋅20 (0⋅33, 4⋅31)	0⋅784
Grades 6–10	0⋅67 (0⋅20, 2⋅22)	0⋅517	0⋅87 (0⋅24, 3⋅09)	0⋅827
Higher education	1		1	
Agriculture land ownership and size (ha)				
No land	0⋅71 (0⋅22, 2⋅26)	0⋅557	0⋅55 (0⋅12, 2⋅49)	0⋅441
<1	0⋅48 (0⋅23, 0⋅96)	0⋅039	1⋅35 (0⋅55, 3⋅33)	0⋅519
1–2	0⋅71 (0⋅37, 1⋅38)	0⋅309	1⋅52 (0⋅66,3⋅53)	0⋅33
>2	1		1	
Family size				
≥7	0⋅73 (0⋅41, 1⋅32)	0⋅3	0⋅99 (0⋅49, 2⋅02)	0⋅995
4–6	0⋅72 (0⋅44, 1⋅18)	0⋅19	0⋅86 (0⋅47, 1⋅55)	0⋅608
≤3	1		1	
Household estimated annual income in ETB				
≤5000	0⋅48 (0⋅23, 1⋅02)	0⋅055	0⋅37 (0⋅16, 0⋅84)	0⋅017
5001–15 000	0⋅61 (0⋅29, 1⋅30)	0⋅204	0⋅47 (0⋅21, 1⋅05)	0⋅066
15 001–30 000	1⋅51 (0⋅66, 3⋅47)	0⋅331	0⋅43 (0⋅17, 1⋅08)	0⋅074
≥30 001	1		1	
Respondents’ religion				
Orthodox/catholic	1⋅84 (0⋅80, 4⋅24)	0⋅15	1⋅96 (0⋅71, 5⋅43)	0⋅196
Muslim	1⋅94 (1⋅03, 3⋅64)	0⋅04	2⋅74 (1⋅35, 5⋅56)	0⋅005
Protestant	1		1	

AOR, adjusted odds ratio; ha, hectare; ETB, Ethiopian Birr.

Maximum variance inflation factor: milk = 2⋅08 and milk products = 2⋅08; pseudo *R*^2^: milk = 0⋅27 and milk products = 0⋅33.

Family size was a factor in poultry and meat consumption patterns. Households with four to six (AOR 0⋅34, *P* 0⋅029) or seven or more (AOR 0⋅11, *P* 0⋅002) members were more likely to have a low frequency of poultry consumption than households with three or fewer members. Family size of seven or more predicted reduced household probability of meat consumption by 62 % (AOR 0⋅38, *P* 0⋅007). An estimated annual income of 5000 ETB or less also reduced meat consumption probability of households by 81 % (AOR 0⋅19, *P* < 0⋅001) compared to those earning more than 30 000 ETB annually (Table 3).

Households with an estimated annual income of 5000 ETB or less (AOR 0⋅38, *P* 0⋅013), four to six family sizes (AOR 0⋅57, *P* 0⋅031) and no chicken ownership (AOR 0⋅33, *P* < 0⋅001) were less likely to consume eggs. Households that did not own donkey were less likely to consume poultry (AOR 0⋅25, *P* 0⋅017), meat (AOR 0⋅38, *P* 0⋅008) and eggs (AOR 0⋅55, *P* 0⋅06) than their counterparts (Table 3).

Table 4 shows that households with more than 2 ha of agricultural land were more likely to report consuming milk on a daily basis than households with 1 ha or less (AOR 0⋅48, *P* 0⋅039). Households who owned cows were more likely to consume milk (AOR 0⋅42, *P* < 0⋅001) and milk products (AOR 0⋅35, *P* < 0⋅001) frequently than households that did not own cows. Households with no cash crop production were more likely (AOR 3⋅01, *P* 0⋅003) to consume milk products more frequently than households producing cash crops. In contrast to households with an estimated annual income of more than 30 000 ETB, those with an annual income of less than 5000 ETB were less likely (AOR 0⋅37, *P* 0⋅017) to consume milk products.

## Discussion

The purpose of the present study was twofold: (1) to assess household-level dietary behaviours related to the consumption of animal products and (2) to identify facilitators, constraints to and determinants of household-level ASF consumption. We found that poultry was not consumed by 92 % of the the households, and meat was not consumed by 60 % of the households over a one-month period. This is in agreement with other papers^(40,41)^ those described limited inclusion of ASF in diets of families in low- and middle-income countries (India, China and Latin America). However, there are studies in which better ASF consumption patterns were reported. In south Ethiopia, once per month consumption of meat was reported for 80, 72 and 26⋅8 % of households in Wolaita Sodo town^(42)^, Mirab Abaya^(43)^ and Hawassa city^(44)^. As the studies were conducted in towns, the difference in consumption practice from the present study could be subject to the availability of retailer butcher houses and households’ interest to pay and purchasing power. The habit of frequent meat consumption in the immediate local culture may also have contributed. A study from Sudan^(45)^ also reported once per week meat consumption frequency for more than two-thirds (68 %) of the studied households. Overall, in the present study, meat consumption was constrained by the reported unavailability (70 %) and unaffordability (80 %) of the food product.

About a quarter of the studied households consumed milk once per day (21 %) or one to two times per week (26 %). According to a study conducted in East Shoa Zone in Ethiopia, 67–100 % of households from urban areas and 67–87 % of households from peri-urban areas consumed milk three to six times per week^(46)^. The variations could be due to study setting, socio-economic and food habit differences in urban and rural scenarios. A study conducted in China identified that urban dwellers consume more animal products than those in rural areas^(47)^. Animal product consumption also increased in response to income growth.

Eggs were consumed one to two times per week (20 %) or one to two times per month (19 %) by about one-fifths of the households during the 1-month period prior to the day of assessment. In the present study, egg consumption was found to be better than poultry, meat and fish. This could be because of relative availability (78 %) and affordability (60 %) of eggs, as many of the participants reported. However, egg consumption frequency in the present study was lower than a report from Hyderabad district in India, where daily egg consumption was reported for half (51 %) of the households^(48)^. Egg consumption habit, higher price as compared to plant-based food items and poultry production primarily for income generation^(49,50)^ might have constrained the consumption of eggs.

Meat was unavailable in the rural settings to more than two-thirds of research participants (70 %), where they usually shop, while 80 % could not afford the price. Plant-based food products were more affordable than meat for 92 % of the respondents. The present findings are supported by reports^(49,51)^ that identified poverty and high price of nutritious foods including animal products as constraints to ASF consumption. A study from Ghana^(52)^ identified low income and lack of market access as contributing factors for low ASF consumption.

Availability, positive attitudes to its quality, relative affordability, less effort needed to prepare and cook, storage and favourable beliefs about health benefits were found to be facilitators to the consumption of eggs. However, income generation priority from poultry rearing and higher price of egg (93 %) as a compared to plant-based food were the constraints identified. A large proportion of households (91 %) in Ethiopia rear chickens for the sake of income generation and savings^(49,50)^ rather than household-level consumption similar to the present findings.

Unlike the findings from the Amhara^(53)^ and Tigray^(49)^ regions of Ethiopia with more Orthodox Christians and from a review on meat consumption^(54)^, religion and related fasting did not hinder the use of ASF in the present study. This could be because of the dominating Protestant (67 %) and Muslim (28 %) religion followers in the present study areas, who do not abstain from the consumption of ASF during their fasting seasons.

Household food insecurity and ASF consumption pattern were associated. Food-insecure households were less likely to consume poultry, meat, eggs, milk and milk products than food-secure households. In Ethiopia, a 40–70 % price increment was documented for nutritious food commodities like eggs, meat, dairy and other products since the year of 2005^(55)^. Studies from Jimma in Ethiopia^(56)^ and Sierra Tarahumara in Mexico^(57)^ identified households’ shifting to inexpensive food items as a coping strategy for food insecurity. This could explain the inverse association between household food insecurity and ASF consumption frequencies observed in the present study districts.

The consumption of ASF is expected to increase when a household owns domestic animals as a source of food commodities and income for diversified diets. According to the results of the present study, households owning cows were likely to consume milk and milk products more frequently than households not owning cows. Households with chickens were also more likely to consume eggs than households with no chickens. Households with donkeys were more likely to consume poultry, meat and eggs than households without donkeys. Studies^(58–61)^ from Ethiopia have identified that donkeys contribute to human livelihood and household food security through their role in income generation and gender empowerment. This might have contributed to the observed positive association between donkey ownership and ASF consumption frequencies.

Lesser farmland size and cash crop production practice were inversely associated with households’ dairy product consumption frequency. Land size is an important asset for farming households in the study districts. Larger land allows households to rear cows on free grazing^(62)^ to serve as a source of dairy products^(63)^. On the other hand, cash crops may constantly take over the limited landholding and make it unfit for traditional livestock farming that would contribute to ASF availability and access.

Education is an important tool for empowering human beings to positively influence livelihood^(64,65)^. In the present study, households with women with better education achievements were more likely to consume eggs than households with women who never attended formal education.

Larger family size has been discussed for its negative effect on household food security^(66,67)^ and that may be because it constrains households’ food purchasing power and challenges intra-household food distribution. Correspondingly, in the present study, more family size was negatively associated with household-level poultry, meat and egg consumption. This may call for food-based interventions on the improvement of diversified agricultural production, family planning and income generation with nutrition education for the public at large.

Less eggs, meat and milk product consumption frequencies were associated with an estimated annual income of 5000 ETB or less. In line with the observed negative association between low income and ASF consumption in the present study, a global analysis on the affordability of dietary recommendations^(68)^ mentioned that diets in low- and middle-income countries might face limitations for nutritious foods like eggs, meat, fish, dairy, fruits and vegetables, as they are high-cost food groups.

Despite comprehensively addressing evidence gaps related to ASF consumption patterns, because the present study is cross-sectional, it cannot identify causal relationships between the identified predictors and respective ASF consumption^(69,70)^. Interpretation of the results must also consider the unavoidable methodologic limitations^(71)^ of consumption frequency assessment like an increased probability of recall bias, as the period for which respondents had to remember was a full month. It is also good to consider the possibility of respondents’ desire to show being needy regardless of status.

## Conclusions

Meat, poultry and fish were consumed less frequently than dairy and eggs. The consumption of poultry, meat and fish was nutritionally negligible. In general, ASF were not consumed often by households in the studied rural districts of Ethiopia. Unavailability, unaffordability, limited ASF consumption tradition, income generation priority from livestock rearing and higher price of ASF than plant-based foods were constraints identified.

Poverty as determined by food insecurity, income and property ownership hampered ASF consumption frequencies. Cow, chicken and donkey ownership amplified selected ASF consumption frequencies. Poultry, meat and eggs were less frequently consumed by households with the lowest estimated annual income category. Women's lack of formal schooling and larger family size also contributed to the less frequent consumption of some types of ASF. Policies and programmes on food-based nutrition interventions should focus on the improvement of diversified agricultural production through nutrition-sensitive agricultural extension, family planning, livelihood improvement, women's empowerment and job opportunities to generate income integrated with nutrition education and evaluate their effects on the nutritional status of the public at general and on ASF consumption patterns in particular.

## Supplementary material

For supplementary material accompanying this paper visit http://dx.doi.org/10.1017/jns.2021.52. click here to view supplementary material
