# Supplementary material for: Determinants and constraints to household-level animal source food consumption in rural communities of Ethiopia
Source: J Nutr Sci. 2021 Aug 9;10:e58. doi: 10.1017/jns.2021.52 (PMC8358840; doi:10.1017/jns.2021.52)
Supplement: Supplementary file 1 [file jnssup.zip › S2048679021000525sup001.docx]

**Table S1** Analysis of facilitators and constraints to the consumption of meat, egg and dairy in Hawassa Milkshed, Ethiopia (n=422)

| **Statement of agreement or disagreement** | **Responses (%)** | | | | | | | | | | | | | | |
| --- | --- | --- | --- | --- | --- | --- | --- | --- | --- | --- | --- | --- | --- | --- | --- |
|  | **Meat** | | | | | **Egg** | | | | | **Dairy** | | | | |
|  | **-2** | **-1** | **0** | **1** | **2** | **-2** | **-1** | **0** | **1** | **2** | **-2** | **-1** | **0** | **1** | **2** |
| The quality of meat/egg/dairy is important for me | 1.2 | 0.5 | 1.9 | 10 | 86.5 | 2.2 | 1 | 6.7 | 8.9 | 81.3 | 0.5 | 0.2 | 1.4 | 7 | 90.9 |
| It is difficult to find meat/egg/dairy that I like or want where I usually shop | 10.9 | 13.8 | 5.7 | 17.7 | 51.9 | 3.4 | 9.6 | 8.1 | 41.6 | 37.2 | 38.7 | 43.1 | 1.4 | 1.3 | 5.5 |
| I am able to afford for meat/egg/dairy | 10.4 | 7.2 | 2.4 | 40.9 | 39.1 | 12.3 | 19.2 | 2.5 | 41.9 | 24.1 | 9.9 | 18.8 | 1.7 | 45.7 | 24 |
| Lack of proper meat/eggs/dairy storage prevents me from eating meat/eggs/dairy as often as I would like to eat | 34.2 | 29.5 | 4.5 | 27.8 | 3.6 | 48.5 | 27.3 | 3.4 | 19 | 1.7 | 41.1 | 34.1 | 3.4 | 20.7 | 0.7 |
| It takes a lot of effort to prepare and cook meat/egg/dairy | 41.1 | 28 | 5.9 | 22.1 | 2.9 | 51 | 26.8 | 6.9 | 14.3 | 1 | 46.9 | 32.5 | 5 | 14.9 | 0.7 |
| I think eating meat/egg/dairy is good for my health | 0.5 | 1.5 | 0.2 | 3.3 | 94.8 | 0.5 | 0.5 | 0.5 | 3.2 | 95.3 | 0.5 | 0.2 | 0.5 | 3.4 | 95.4 |
| My health doesn't allow me to eat meat/egg/dairy | 79.8 | 9.3 | 3.6 | 4.8 | 2.6 | 83.3 | 7.6 | 2.5 | 7.6 | 83.3 | 82.5 | 9.4 | 2.4 | 2.9 | 2.9 |
| Meat/egg/dairy was a regular component of my diet while growing up | 25.7 | 24.2 | 2.9 | 28.5 | 18.8 | 19.7 | 21.7 | 4.4 | 31.8 | 22.4 | 9.6 | 12.7 | 1.7 | 37.5 | 38.5 |
| My religion doesn't prohibit me eating of meat/egg/dairy | 1.5 | 2.5 | 4.5 | 9 | 82.4 | 0.9 | 1.1 | 5.9 | 8.4 | 83 | 0.8 | 0.4 | 5.8 | 8.2 | 84.9 |
| I don't eat some kinds of meat because of my religion | 44.4 | 10.9 | 6.2 | 29.9 | 8.6 | - | - | - | - | - | - | - | - | - | - |
| I don't eat meat/egg/dairy when I am fasting | 83.4 | 8.8 | 1.4 | 2.1 | 4.3 | 87.9 | 5.7 | 1.7 | 1.7 | 3 | 87.5 | 6 | 1 | 2.4 | 3.1 |
| Although we rare chicken and other livestock, we use them for income | 23.2 | 15 | 26.7 | 20.3 | 14.8 | 29.6 | 17.2 | 23.4 | 20.4 | 9.4 | 37.5 | 20.4 | 22.8 | 11.8 | 7.5 |
| Eating plant-based foods is more affordable to us than eating of meat/egg/dairy | 1.9 | 2.1 | 4.3 | 13.5 | 78.1 | 2.5 | 1.2 | 3 | 22.7 | 70.7 | 4.1 | 3.9 | 2.4 | 18.1 | 71.6 |

2: Strongly Agree; 1: Slightly Agree; 0: Neither Agree nor Disagree; -1: Slightly Disagree; -2: Strongly Disagree
