# Supplementary material for: Determinants and constraints to household-level animal source food consumption in rural communities of Ethiopia
Source: J Nutr Sci. 2021 Aug 9;10:e58. doi: 10.1017/jns.2021.52 (PMC8358840; doi:10.1017/jns.2021.52)
Supplement: Supplementary file 1 [file jnssup.zip › S2048679021000525sup002.docx]

| **Statement of agreement or disagreement** | Response (%) | | | | |
| --- | --- | --- | --- | --- | --- |
|  | **-2** | **-1** | **0** | **1** | **2** |
| The quality of fish is important to me | 0.4 | 16.2 | 35.3 | 16.2 | 47 |
| The fish that I want to eat are available where I usually shop | 0.4 | 0.8 | 0.3 | 29.7 | 68.8 |
| I am able to afford fish | 5.3 | 21.1 | 15.5 | 33.5 | 24.4 |
| It takes a lot of effort to prepare and cook fish | 5.3 | 25.9 | 35.7 | 18.8 | 14.3 |
| Lack of proper fish storage prevents me from eating fish as often as I would like to eat | 3 | 25.9 | 34.8 | 19.9 | 16.3 |
| I think eating fish is good for my health | 2.6 | 0.3 | 21.1 | 0.4 | 75.6 |
| My health does not allow me to eat fish | 4.9 | 1.9 | 47.8 | 7.1 | 38.3 |
| Fish was a regular component of my diet while growing up | 35.3 | 21.4 | 26.5 | 9.2 | 7.5 |
| My religion doesn't prohibit me eating of fish | 1.5 | 0.4 | 7.1 | 17.7 | 73.3 |
| I don't eat fish when I am fasting | 1.5 | 1.5 | 12.4 | 8.6 | 75.9 |
| Although we rear fish, we sell them for income | - | - | - | - | - |
| Eating plant based foods is more affordable to us than eating of fish | 0.8 | 0.3 | 7.9 | 10.2 | 80.8 |

**Table S2** Analysis of facilitators and constraints to the consumption of fish in Hawassa Milkshed, Ethiopia (n=422)

2: Strongly Agree; 1: Slightly Agree; 0: Neither Agree nor Disagree; -1: Slightly Disagree; -2: Strongly Disagree
